# The Single-Knot Running Vesicourethral Anastomosis after Minimally Invasive Prostatectomy: Review of the Technique and Its Modifications, Tips, and Pitfalls

**DOI:** 10.1155/2016/1481727

**Published:** 2016-05-31

**Authors:** Simone Albisinni, Fouad Aoun, Alexandre Peltier, Roland van Velthoven

**Affiliations:** Department of Urology, Institut Jules Bordet, Université Libre de Bruxelles, Boulevard de Waterloo 121, 1000 Brussels, Belgium

## Abstract

The vesicourethral anastomosis represents a step of major difficulty at the end of minimally invasive radical prostatectomy. Over 10 years ago, we have devised the single-knot running vesicourethral anastomosis, which has been widely adopted in urologic departments worldwide. Aim of the current paper is to review the technique, its adaptability in complex situations, its complications, and possible modifications, including the use of barbed sutures.

## 1. Introduction

Minimally invasive radical prostatectomy is today one of the most common procedures in urologic oncology. In the last years, the introduction of the robotic platform has increased exponentially the amount of cases performed, given the increased maneuverability of the surgical robot and the more intuitive manipulation compared to standard laparoscopy. Nonetheless, at the end of a complicated and highly detailed procedure as radical prostatectomy, the vesicourethral anastomosis (VUA) represents a challenge for every urologist [1, 2]. Moreover, the functional consequences of an imperfect anastomosis may be serious and lead to dramatic consequences on the patients' quality of life, even in case of an oncologically impeccable surgery. For these reasons, this reconstructive surgical step must be mastered by any urologist wanting to perform minimally invasive prostatectomy.

Over 10 years ago we introduced a single-knot running VUA, today more known as the van Velthoven technique [3]. This technique requires only one intracorporeal knot, allows a water-tight approximation of the bladder neck to the urethra, and is easily teachable. Currently, urologists worldwide have adopted such technique, although many variations exist [4–6]. Aim of the current paper is to review the surgical technique and analyze its complications and its adaptability to complex situations.

## 2. The Single-Knot Running Vesicourethral Technique

In order to perform a valid vesicourethral anastomosis, there are some key points which the surgeon should always keep in mind: first, the anastomosis must be tension-free and allow a precise alignment between the bladder neck and the urethral stump. Second, the suture should be nonischemic and waterproof, to prevent urinary leakage and avoid postoperative strictures. Third, the method should be easy to learn and of rapid execution. The success of a vesicourethral anastomosis therefore depends upon meticulous attention to detail and the optimization of technical factors that affect anastomotic integrity. The single-knot running vesicourethral anastomosis respects these principles and has become a common method of reconstruction [3].

The technique begins by inspecting the bladder neck with careful attention to ureteral orifices. The degree of bladder neck preservation will dictate the length of the suture. It varies accordingly between 12 cm and 20 cm. The running suture is prepared extracorporeally by tying together the two ends of twin dyed sutures of 3-0 Monocryl RB-1 (Ethicon, USA). The running stitch is initiated by placing both needles outside-in through the bladder neck and inside-out on the urethra, one needle at the 5:30-o'clock position and the other needle at the 6:30-o'clock position. The sutures are run from the 6:30 and 5:30-o'clock positions toward the 9:00 and 3:00-o'clock positions, respectively. After two throws through the urethra and three throws through the bladder are completed, the sutures are cinched down with gentle traction on each thread simultaneously or alternately, bringing the bladder neck as a unit tightly into position with the urethra. This approximation provides a secure posterior wall with no gap visualised between sutures and allows a Foley catheter to be placed into the bladder. The symmetric loops act as a block and pulley mechanism, thereby enabling approximation of the dorsal part of the anastomosis to be carried out without tension or traction. The sutures are now both passed inside-out on the urethra, in order to perform then a U-turn, by going outside-in on the urethra at the 10:00 and 2:00 positions. This transition suture allows the stitch to exit the bladder on its outer surface, which is more resistant to traction compared to the frail and delicate urethra. The sutures are continued to the 12:00-o'clock position and tied to each other. This intracorporeal knot now, like the initial extracorporeal knot, rests on the exterior of the bladder. Although the robotic platform has greatly eased the throw of stiches, the single-knot technique remains easy and effective, given the need to place only one stich. In cases where a large bladder neck exists, with a consequent mismatch between the bladder and the urethra, a posterior or anterior tennis-racket can be performed. This can also help if the ureteral orifices are too close. Generally, we leave the Foley catheter in place for 5 days and remove it after a retrograde cystogram showing no leakage [7].

Different techniques have been described for minimally invasive VUA, notably interrupted sutures, and other modifications of the running suture technique. Bai et al. compared the single-knot running technique to interrupted sutures and a modified interrupted technique, all performed by laparoscopy [8]. The single-knot technique was associated with a reduced operative time (median 15 min) compared to the interrupted sutures, indeed as a consequence of the single knot needed. The reduction in requirement for knot-tying may enable surgeons with limited suturing experience to master this difficult technical step, unavoidably located at the end of a challenging procedure. This time-gain is true also in robotic procedures, although the robotic platform greatly simplifies the task by its intuitive manipulation. Furthermore, it is important to recognize that, to perform interrupted sutures, two needle drivers are fundamental. In the single-knot technique instead, only one needle drive can be used in combination with bipolar fenestrated or Maryland forceps in the nondominant hand: in robotic procedures this can allow a saving of approximately €220 per procedure and it is how we are currently performing VUA in our department.

Recently, we have published the results of an international survey evaluating the acceptance and appreciation of urologists worldwide of the single-knot running VUA, over ten years after its introduction [7]. 391 urologists participated in a total of over 120 000 minimally invasive prostatectomies. Overall the survey showed that the single-knot running technique is an accepted and commonly used procedure to perform vesicourethral anastomosis during laparoscopic and robotic-assisted urologic surgery. In particular, a low rate (<2%) of technical problems was encountered in the experience of the survey participants, as well as a low rate of early (<2% of urinary leakage) and late complications (<2% of anastomotic strictures). Catheter times were reported to be generally of 5 days after performing this technique. Moreover, 45% of the respondents found that this is a technique which “belongs to the commonly used procedures in urology.”

## 3. Complex Situations

Minimally invasive radical prostatectomy is always challenging. However, there are particular situations in which it can make the situation particularly complex, even for experienced operators. In particular, major challenges can arise when radical prostatectomy is performed after another primary prostate cancer treatment (i.e., radiation therapy, HIFU, and cryoablation), or after prostate surgery for benign prostatic enlargement. The complexity of the dissection, the tissue fibrosis, may lead to an excessive bladder neck opening, with a consequent discordance between the bladder and the urethra. We frequently face these situations by performing an anterior tennis racket reconstruction, which obviates excessive suturing of bladder tissue to the urethra, thus limiting the risk of anastomotic leakage. Moreover, if there has been exposure to radiation or thermal energy, tissue healing is usually impaired and the risk of postoperative complications is therefore elevated: Ouzaid et al. retrospectively analyzed 2215 patients undergoing minimally invasive radical prostatectomy with the van Velthoven technique. After a median follow-up of 43 months, anastomotic strictures occurred in 30 (1.4%) patients, and both previous radiotherapy and previous transurethral resection of the prostate (TURP) were significant predictors of these complications [9]. Series describing functional outcomes of salvage RALP after primary treatment (radiation, brachytherapy, and HIFU) confirm that complications following such procedure are frequent [10–14]: Yuh et al., analyzing complications of salvage RALP in 51 men with recurrent prostate cancer, found an 18% anastomotic leakage rate and found that 16% of patients developed an anastomotic stricture [10]. Eandi et al. also reported outcomes in 18 men undergoing salvage RALP: 33% of patients had a leak requiring prolonged drainage (mean 38 days), and 17% developed an anastomotic stricture [11]. The surgeon should keep this in mind while performing the suture in these complicated cases. We recommend additional catheter days in these patients, in order to allow full tissue healing before applying tension on the anastomosis.

Similarly after benign prostatic enlargement surgery, the VUA is particularly challenging. There are a number of factors to take into account: the modified anatomy of the vesicoprostatic junction, the increased thickness of the bladder wall, increased periprostatic adhesions, and difficulties in identifying the ureteral orifices. A study evaluating 26 men undergoing RALP after previous TURP confirmed these difficulties. The authors reported not only increased peroperative difficulties (which was reflected in increased blood losses and increased conversion to open surgery), but also worst postoperative functional outcomes: a prolonged urine leakage and an anastomotic stricture were found in 11.5% and 14% of men in the post-TURP group, respectively [15].

## 4. Barbed Sutures

In the discovery years of laparoscopic prostatectomy, the VUA was indeed a wearing and extremely complicated surgical step, aggravated by its inevitable placement at the end of the procedure. In our first report, median time was 35 minutes, although experienced laparoscopic surgeons were performing the anastomoses. Today, with experience, robotic technology, and divulgation of techniques, VUA has become an easier task which usually requires about 15 mins in expert hands and 30 mins for novice surgeons [2, 6, 16–18]. In the initial description of the technique, we use two 3/0 poliglecaprone-25 absorbable monofilament sutures (Monocryl*™*) tied at their end, and these are the sutures which we still use today, as they have excellent manageability, good distribution of tension, and favorable resorption characteristics. Nonetheless, in recent years, barbed sutures have begun to be divulgated in both laparoscopy and open surgery. Their characteristic is the presence of small barbs which allow the suture to be pulled in one direction, without being able to be pulled back. As a consequence, these sutures do not lose tension once pulled and as such do not lose traction even if left free.

Several types of barbed sutures exist. V-LOC*™*, commercialized by Covidien, is characterized by a high density of barbs, 20 per centimeter, and unidirectionality: at the end of the suture is a small loop which serves to lock the suture after the first throw. As such, when used for VUA, two V-LOC devices are used and locked together at their ends. V-LOC is designed such that the first 3 centimeters of thread after the needle is nonbarbed: this allows the surgeon to undo a stitch if it is misplaced. V-LOC is available in 2/0, 3/0, and 4/0, with two types of absorbable materials (90 and 180, referring, resp., to the average absorption time) and one nonabsorbable texture. Quill*™*, by Angiotech Pharmaceutics, is a barbed suture available either in uni- or bidirectional form: the bidirectional suture has 2 needles at its ends, with barbs oriented in opposite direction in the two halves of the suture, starting from a transition point at the center. Quill sutures have 8 barbs per centimeter, disposed in a helical pattern to allow equal distribution of strength. Stratafix*™*, produced by Ethicon, is another option across barbed sutures. Its design is characterized by a helical disposition of barbs, the possibility to have uni- or bidirectional sutures, and a vast choice in suture width, length, material, and needle type. The equivalence in biocompatibility and strength of barbed sutures compared to standard sutures has been demonstrated in different animal studies [19, 20], making these sutures a possible option for VUA.

Surgeons have throughout fully evaluated barbed sutures in the context of VUA, analyzing whether a real clinical advantage in terms of operative time and functional results was determined by these sutures compared to standard absorbable monofilaments. Specifically, Tewari et al. compared V-LOC to Monocryl in VUA in 100 men. They reported a significantly reduced anastomotic time with V-LOC, although this difference was ~5 min and as such its clinical significance is questionable [16]. Similarly, Moran et al. tested a bidirectional barbed suture (Quill), comparing it to Monocryl, finding minimal differences in time and in surgeon security score [21]: although the Quill suture was faster to deploy (17.3 versus 19.2 minutes), and the security score by the surgeon was greater, also in this case it is important to bear in mind clinical and not only statistical differences. Sammon et al. randomized 64 men undergoing robotic prostatectomy to have the VUA performed with a barbed versus a standard monofilament: no significant differences in urinary leakages and late bladder neck contractures were found across the two arms of the study [22]. Hemal et al. compared V-LOC to Monocryl, reporting shorter operative times (8 versus 14 min) and higher surgeon comfort with the V-LOC [1]. Zorn et al. in a prospective randomized trial comparing Monocryl versus V-LOC, after a median follow-up of 6.2 months, neither leakage nor anastomotic stricture was observed in the V-LOC group, and continence rates were similar across the two groups (88 versus 92%, *p* = 0.70) [23]. Similarly, Massoud et al. compared VUA performed with interrupted Vicryl stiches to VUA with a continuous V-LOC suture [24]. No difference in anastomotic stricture rate requiring internal urethrotomy (2.5% in each group) was found across the two groups. Moreover, at 12-month follow-up, comparable continence rates were found in the two arms (97.5% with V-LOC versus 95% with interrupted sutures, *p* = 0.368).

The two major concerns associated with barbed sutures are costs and the possibility of increased tissue inflammation leading to late complications after VUA. Indeed barbed sutures are more expensive than standard threads: in Europe, a V-LOC suture costs around 17€, making VUA material cost approximately 34€, compared to 7€, which is roughly the cost of two Monocryl 3/0 sutures. Although this may seem negligible compared to the cost of the entire procedure, we are in a period of economic crisis and efforts should be made to reduce costs. Regarding tissue inflammation, there is no evidence that barbed sutures determine its increase [25, 26]. Nonetheless, long-term functional outcomes after barbed-suture VUA are awaited, albeit encouraging short-term results [8, 16, 27, 28].

## 5. Complications of the Single-Knot Running VUA

Every surgical procedure has its complications, which will sometimes inevitably occur, even to the most experienced operator. As such, it is of uttermost importance to be familiar with these complications, keep them in mind while performing the anastomosis, and do everything possible to avoid their manifestation.

An anastomotic leak is short-term complication following vesicourethral anastomosis and is associated with significant morbidity including postoperative ileus, infection, metabolic abnormalities, prolonged hospital stay, and urinoma formation with potential risk of anastomotic disruption. After performing a single-knot running VUA, the incidence of anastomotic leakages varies ranging from 0 to 7.5% [1, 29, 30]: compared to other techniques, investigators have found that a single-knot running technique reduces the rate of leakages [2, 17]. Nonetheless, the consequences of such leakages on the long term remain controversial. Although some authors report no statistical impact of leakage on subsequent stricture formation [2, 31, 32], Surya et al. suggested that prolonged urine leaks are a risk factor for such late complication [33]. The investigators hypothesized that the fibrotic healing process of the leakage itself could lead to excessive scarring and consequent stricture formation [1, 2, 17, 29–32].

Concerning long-term complications, anastomotic strictures can occur after VUA and generally require surgical correction. Significant morbidity may be associated with the development of an anastomotic stricture, including infection, urinary retention, the need for additional invasive surgery, and future incontinence [34]. The pathophysiology of postoperative stricture formation remains poorly understood, although it is clear that both patient- and surgery-related factors exist [35]. Within the patient-related factors, smoking, diabetes, hypertension, obesity, chronic renal insufficiency, and coronary artery disease are all associated with stricture in large scale observational studies [36]. Regarding the surgical technique, tissue-ischemia, excessive narrowing of the anastomosis, and/or lack of mucosal apposition at the time of the procedure are known risk factors [36, 37]. Finally, postoperative radiation is another well-known cause of anastomotic stricture by inducing ischemia and fibrosis [9, 36, 37]. Most surgical series in which the single-knot method was used to perform VUA report stricture rates ranging from 0 to 3% [1, 2, 28–30, 38]. These positive findings are a consequence of the wide end-to-end mucosal approximation, the even distribution of tension due to the pulley mechanism (funneling parachute), and a consequent reduced tissue-ischemia.

## 6. Conclusions

In conclusion, over 10 years has passed since the introduction of the single-knot running VUA technique and today this is a valid and safe technique for VUA. Although many excellent variations exist [4–6], urologists involved in minimally invasive surgery should be familiar with the technique and its tips, tricks, and pitfalls, in order to avoid complications and obtain the best functional results.
